# An Automated Approach for Electric Network Frequency Estimation in Static and Non-Static Digital Video Recordings

**DOI:** 10.3390/jimaging7100202

**Published:** 2021-10-02

**Authors:** Georgios Karantaidis, Constantine Kotropoulos

**Affiliations:** Department of Informatics, School of Sciences, Aristotle University of Thessaloniki, 54124 Thessaloniki, Greece; costas@csd.auth.gr

**Keywords:** estimation by rotational invariant techniques (ESPRIT), short-time Fourier transform (STFT), multiple signal classification (MUSIC), simple linear iterative clustering (SLIC), video forensics

## Abstract

Electric Network Frequency (ENF) is embedded in multimedia recordings if the recordings are captured with a device connected to power mains or placed near the power mains. It is exploited as a tool for multimedia authentication. ENF fluctuates stochastically around its nominal frequency at 50/60 Hz. In indoor environments, luminance variations captured by video recordings can also be exploited for ENF estimation. However, the various textures and different levels of shadow and luminance hinder ENF estimation in static and non-static video, making it a non-trivial problem. To address this problem, a novel automated approach is proposed for ENF estimation in static and non-static digital video recordings. The proposed approach is based on the exploitation of areas with similar characteristics in each video frame. These areas, called superpixels, have a mean intensity that exceeds a specific threshold. The performance of the proposed approach is tested on various videos of real-life scenarios that resemble surveillance from security cameras. These videos are of escalating difficulty and span recordings from static ones to recordings, which exhibit continuous motion. The maximum correlation coefficient is employed to measure the accuracy of ENF estimation against the ground truth signal. Experimental results show that the proposed approach improves ENF estimation against the state-of-the-art, yielding statistically significant accuracy improvements.

## 1. Introduction

The vast amount of information contained in multimedia content, i.e., audio, image, and video recordings, has prompted perpetrators to commit forgery attacks distorting the digital content. Digital forensics advancements have experienced an exponential growth in the last decades, as digital manipulation methods are constantly evolving and affecting various aspects of social and economic life. To this end, emphasis has been put on advancing emerging technologies in the field of digital forensics, which can efficiently verify the authenticity of multimedia content and cope with multimedia forgeries. A comprehensive survey of image forensics techniques can be found in [1].

In recent years, the Electric Network Frequency (ENF) has been employed as a tool in forensic applications. The ENF is a time-varying signal, which fluctuates around its nominal frequency, i.e., 50 Hz in Europe and 60 Hz in the United States. These fluctuations are due to the instantaneous load differences of the power network (i.e., the power grid). They exhibit an identical trend within the same interconnected network. The ENF is a non-periodic signal, which can act as a fingerprint for digital forensics applications [2]. It can be embedded in digital audio recorded by devices plugged into the power mains or by devices placed near the electric outlets and power cables. The ENF can be captured in video recorded in indoor environments due to fluorescent light. Illumination intensity variations resemble ENF variations in the power grid [3]. Thus, ENF estimation can be exploited for multimedia authentication, timestamp verification, and forgery detection in audio and video recordings. Until recently, the research has mainly been focused on audio recordings, where many advances have been achieved.

To begin with, let us briefly survey ENF estimation in audio recordings, because the same ENF estimation methods are also applied to a one-dimensional (1D) time-series extracted from video recordings. A comprehensive study addressing the ENF detection problem was presented in [4], where many practical detectors were introduced. The detectors were shown to have a reliable performance in relatively short recordings, enabling accurate ENF detection in real-world forensic applications. An alternative to the conventional Short-Time Fourier Transform (STFT) is advanced spectral estimation [5], offering high-resolution at the expense of increased computational complexity. For example, an iterative adaptive approach accompanied by a dynamic programming was applied to frequency tracking. An optimized maximum-likelihood estimator for ENF estimation was proposed by employing a multi-tone harmonic model [6]. Multiple harmonics were combined to provide a more accurate estimation of the ENF signal and the Cramer–Rao bound was used to bound the variance of the proposed estimator. Following the same reasoning, a spectral estimation approach was presented in [7], combining the ENF at multiple harmonics. Each harmonic was weighted depending on its signal-to-noise (SNR) ratio. A pre-processing approach was proposed in [8] that was based on robust principal component analysis to reduce noise interference and to enable accurate ENF estimation. There, a weighted linear prediction approach was also employed for ENF estimation. In [9], a lag window was designed to offer an optimal trade-off between smearing and leakage by maximizing the relative energy in the main lobe of the window. It was incorporated in the Blackman–Tukey method, offering accurate ENF estimation with low computational requirements. A Fourier-based algorithm for high-resolution frequency estimation was introduced in [10]. Specific spectral lines were taken into consideration instead of the entire frequency band. In [11], a comprehensive study of the parameters that affect ENF estimation accuracy was undertaken. In the pre-processing stage, signal filtering and temporal window choice were found to be critical in delivering accurate estimation results. A fast version of Capon spectral estimator based on Gohberg–Semencul factorization was presented in [12]. That method along with the use of a Parzen temporal window led to accurate ENF estimation. To address the problem of noise and interference, frequency demodulation was employed for ENF estimation [13]. Several high-resolution frequency estimation methods were discussed in [14]. That work aimed to achieve high performance and to maintain low computational complexity by using as few samples per frame as possible. An integrated and automated scheme for ENF estimation was developed in [15]. A framework for ENF estimation from real-world audio recordings was presented in [16]. First, signal enhancement was proposed, which was based on harmonic filtering. Second, graph-based harmonic selection was elaborated. In [17], a unified approach was proposed to detect multiple weak frequency components under low SNR conditions. Iterative dynamic programming and adaptive trace compensation were employed to identify the frequency components. A multi-tone model for ENF detection applied prior to ENF estimation was presented in [18].

The ENF can also be exploited to detect tampering in multimedia recordings. An edit detection approach taking advantage of the time-varying nature of ENF was proposed in [19]. Multimedia authentication was formulated as a problem of phase change analysis employing the Fourier Transform in [20]. An audio verification system for tamper detection and timestamp verification was proposed in [21]. The system employed absolute-error-maps. A tamper detection framework based on support vector machines was introduced in [22]. That framework exploited abnormal ENF variations caused by tampered regions.

In [23], it was demonstrated that the ENF can be exploited to determine the location of recordings even if they are captured within the same interconnected grid. A multi-class machine learning system was proposed to identify region-of-recordings in [24]. It took advantage of features related to ENF differences among power grids without the need for a reference ENF signal. A convolution neural network system was tested for identifying audio recordings that have been recaptured in [25]. The system worked properly for very short audio clips and was able to combine both the fundamental ENF and its harmonics. To cope with noise interference, a filtering algorithm was introduced in [26]. It employed a kernel function to create a time–frequency representation facilitating ENF estimation. The existence of reliable ENF reference databases is critical for multimedia authentication applications. A method to create ENF reference databases based on geographical information systems (GIS) was presented in [27]. Recently, ENF was explored as a tool for device identification [28]. The proposed method was based on the analysis of harmonic amplitude coefficients, which were employed to deliver an accurate identification of acquisition devices. The ENF is a stochastic signal and its values depend on various exogenous and endogenous factors. In [29], a study was carried out on the factors affecting the capture of ENF in audio recordings as well as on the impact of the audio characteristics.

Although significant attention has been paid to ENF estimation in audio recordings, it was found that the ENF can also be traced in video recordings. The ENF can be estimated in videos captured under the illumination of fluorescent bulbs in indoor environments [3]. ENF variations caused by power grid networks affect the illumination intensity, and each frame captures a time-snapshot of ENF. ENF video estimation approaches can be divided into two categories based on the recording sensor type. The first category consists of videos captured by charge-coupled device (CCD) sensors, which employ a global shutter mechanism. This type of sensor instantly captures all pixels of a frame. Thus, each frame depicts a specific time snapshot. When CCD sensors are used, the state-of-the-art approach for ENF estimation is based on averaging all pixels in each frame of static videos [3]. For non-static videos, state-of-the-art ENF estimation suggests averaging all steady pixels in each video frame. The second category consists of videos captured by complementary metal oxide semiconductor (CMOS) sensors. Such sensors employ a rolling shutter mechanism, which acquires a row at a time in each video frame [3,30]. A comprehensive analysis of the rolling shutter effect was conducted in [31]. An analytical model for videos captured using a rolling shutter mechanism was developed, demonstrating the relation between ENF variations and the idle period length. ENF-based video forensics are not trivial, especially for non-static video recordings. ENF presence detection based on superpixels (i.e., multiple pixels) was proposed in [32]. The proposed approach could be applied to static and non-static videos captured by both CCD and CMOS camera sensors. Recently, a method for ENF estimation in non-static videos was presented in [33]. This method could be accurately utilized in video recordings whose frame rate is unknown. The ENF was applied to video recordings for camera identification in [34]. Video synchronization can be efficiently achieved by employing the ENF. Video synchronization methods were developed in [35,36] that were based on ENF signal alignment. A forgery detection algorithm based on ENF signal was proposed in [37] without needing any ground truth signal. A technique to detect false frame injection attacks in video recordings using the ENF was discussed in [38]. ENF was employed to authenticate video feeds from surveillance cameras. ENF estimation and detection in single images captured by CMOS camera sensors constitutes a challenging task. Novel investigations taking into consideration the ENF strength were described in [39]. ENF estimation in videos with a rolling shutter mechanism was presented in [40]. Both parametric and non-parametric spectral estimation methods were combined for accurate ENF estimation.

In this paper, inspired by [32], an automated approach is proposed for ENF estimation from CCD video recordings based on Simple Linear Iterative Clustering (SLIC) [41]. Areas of common characteristics that include superpixels are generated using the SLIC algorithm. The proposed approach takes into consideration only the superpixels whose average intensity exceeds a predefined threshold. It is shown that within these areas, the embedded ENF is not hindered by any interference, resulting in more accurate estimation regardless of whether the video recording is static or not. The novelty of the proposed approach lies in (1) the creation of areas with similar characteristics and (2) the estimation of ENF exploiting only these areas in contrast to what has been achieved for ENF estimation in videos so far. The motivation for the development of the proposed approach is to mitigate the interference and noise caused by textures, shadows, and brightness that are present in real-life applications, such as surveillance videos. By doing so, we advance the related literature, where static videos are mostly used, such as the “white wall” recordings. From a practical point of view, the proposed approach enables automated ENF estimation regardless of whether the video recording is static or non-static. Thus, it can be applied to practical forensics applications, such as multimedia content authentication, indicating the place where a recording was captured, and revealing the time the recording was made. It is worth noting that the proposed approach is tested on real-world static and non-static videos of escalating difficulty in order to simulate real conditions. The maximum correlation coefficient (MCC) between the estimated ENF and the reference signal is employed to measure ENF estimation accuracy. Moreover, hypothesis testing is performed to assess the statistical significance of the improvements delivered by the proposed approach.

The remainder of the paper is organized as follows. Section 2 details ENF fundamentals, and Section 3 presents the proposed approach; Section 4 describes the dataset and discusses the derived results; conclusions, limitations, and future research are drawn in Section 5.

## 2. ENF Fundamentals

The ENF was initially introduced by C. Grigoras [2,42] to attest to the authenticity of digital recordings, to determine the time they were recorded, and to indicate the area they were captured. In particular, when it comes to video recordings, ENF estimation can determine whether the multimedia content has undergone major alterations. Moreover, ENF can reveal the area where the indoor video was recorded. When the estimated ENF is compared against a reference ground truth, the time the video was recorded is revealed. The proposed approach aims at improving ENF estimation, whose practical applications fall into forensic science. The importance of ENF is due to its unique properties, which makes it a powerful tool in forensic applications. Once the ENF signal has been estimated, a comparison against a reference ENF database should be made in order to assess estimation accuracy.

The most remarkable properties of the ENF signal are summarized as follows:

- The ENF is a non-periodic signal randomly fluctuating around the fundamental frequency.
- ENF fluctuations are identical within the same interconnected network.
- The ENF signal can also be found in higher harmonics [43].

Many approaches have been proposed to efficiently estimate ENF depending on the particularities of each recording.

### 2.1. ENF Estimation

The ENF is embedded in the electric light signal. Assuming stationarity within short-time segments of the signal, the ENF is modeled as

(1) s(t)=Asin(2πft+ϕ)

where *f* is the fluctuating frequency representing the ENF component, *A* is the signal magnitude, and ϕ corresponds to signal phase. There are more complex ENF models, such as that proposed in [44].

It has been shown recently that ENF traces can be embedded in video recordings due to light intensity variations. Such recordings are captured in the presence of fluorescent light or the light emitted by incandescent bulbs [35]. The light intensity is directly connected to electric current and its nominal frequency is influenced by the ENF signal, fluctuating at twice the nominal frequency of ENF, i.e., 100 Hz in Europe, and 120 Hz in the United States. The lower temporal sampling rate of cameras capturing video recordings compared to frequency components in light flickering results in a significant aliasing of ENF signals. Thus, ENF is present at different frequencies than those appearing in audio recordings. These frequencies can be derived by applying the sampling theorem [45]. In addition to the fundamental frequency of power mains, it is the frame rate of video camera that influences the aliased base frequency of ENF in video recordings [3]. The aliased frequency $f_{E}$ emanated from fluorescent illumination is given as follows [46]:

(2) $$f_{E}=\left|f_{l}-\gamma f_{s}\right|\leq \frac{f_{s}}{2}$$

where $f_{s}$ denotes the sampling frequency of camera, $f_{l}$ denotes the frequency of light source illumination, and γ denotes an integer. Aliased frequencies of ENF based on different camera frame rates and power main frequencies are listed in Table 1.

The ENF estimation procedure in video recordings differs slightly from that employed in audio ones. The difference is in the pre-processing stage. Two cases are examined depending on whether the video recordings are static or non-static. Regarding static videos, the state-of-the-art [3] suggests computing the mean intensity of each frame, transforming the two-dimensional (2D) images into a 1D time-series. It is worth noting that the majority of experiments conducted so far employ static recordings of white wall videos. Here, we employ a variety of static recordings different than white wall videos, as detailed in Section 4.1. Regarding non-static videos, the current practice is to compute the mean intensity of relatively stationary areas of each frame. In both categories, a 1D time-series is formed and the estimation procedure follows that employed for audio recordings. This time-series is treated as a raw signal that is passed through a zero-phase bandpass filter around the frequencies where ENF appears. Specifically, the bandpass edges of the filter are set at 9.9 and 10.1 Hz when the nominal frame rate is 30 Hz despite the fact that the nominal frame rate was claimed to be 29.97 Hz in [33]. The bandpass edges employed herein accommodate also the aliased base frequency, which corresponds to a nominal frame rate of 29.97 Hz. The filtering procedure is of crucial importance in ENF estimation [11]. Subsequently, the signal is split into *V* overlapping segments of *L* samples size. Each segment is shifted by *S* s from its immediate predecessor and is multiplied by an *L*-size rectangular window. Any temporal window can be employed in the pre-processing procedure. Afterward, the prevalent frequency of each segment is estimated by spectral estimation. Frequently, a quadratic interpolation is used to overcome the interference that hinders the entire procedure and results in more precise ENF estimation [5,9]. Here, the estimated ENF signal f is calculated by employing shifts of 1 s (i.e., S=1).

## 3. Proposed Method

Here, a video ENF estimation approach for static and non-static video recordings is proposed. It is based on the SLIC algorithm for image segmentation. The SLIC algorithm generates superpixels, which are regions of similar characteristics. The idea behind the proposed approach is that in regions having high luminance levels and not hindered by shadows or dark areas, light source variations can easily be detected, and thus, the ENF signal can be estimated more accurately. The first step of the proposed approach generates *N* regions with similar characteristics in the first frame of a video recording. Afterward, the mean intensity values $\zeta_{n}\left(1\right)$, n=1,2,…,N of all regions in the first frame are computed and only those exceeding a predefined threshold τ are retained. Let ζ(1) be the vector with elements $\zeta_{n}\left(1\right)$. If $\bar{N}=\left|\left\{n:\zeta_{n}\left(1\right)>\tau \right\}\right|$ denotes the size of region mean intensity values exceeding the threshold, then the mean intensity value for the first frame is given as follows:

(3) $$x\left(1\right)=\frac{1}{\bar{N}}\sum_{n=1}^{N}\zeta_{n}\left(1\right)u\left(\zeta_{n}\left(1\right)-\tau \right)$$

where $u(\zeta_{n}\left(1\right)-\tau )$ denotes the Heaviside function.

In the next step, the generated regions from the first frame are located in all Λ frames of the video recording. For a video recording with a duration of 12 min, Λ= 21,600 frames. Employing these regions, the mean intensity values of the regions are computed and, then, the mean intensity value in each frame is calculated, as in (3). In this way, each video frame is represented by an intensity value x(t), t=1,2,…,Λ.

A non-parametric, namely the STFT, and a parametric method, i.e., the Estimation by Rotational Invariant Techniques (ESPRIT), were employed for ENF estimation. Hereafter, the frames, indexed by *t*, will be referred to as samples.

The STFT is one of the most common methods in time-frequency analysis of signals. Assuming stationary within the short-time segments of the signal, the Discrete-Time Fourier transform is computed for each time segment [47]:

(4) $$X_{l}\left(\omega \right)=\sum_{t=-\infty }^{\infty }x\left(t\right)w\left(t-lG\right)e^{-j\omega t}$$

where w(t) denotes a window function of length *L*, $X_{l}\left(\omega \right)$ is the discrete-time Fourier transform of the windowed data centered around lG, and $G=S\;f_{s}$ is the hop size in samples. The proper selection of window function constitutes a very important issue in STFT and, generally, in the majority of time–frequency analysis methods. This is because an optimal trade-off between time and frequency resolution is sought. Let ${\hat{\phi }}_{l}\left(\omega_{\kappa }\right)\propto {\left|X_{l}\left(\omega_{\kappa }\right)\right|}^{2}$ be the periodogram of the $L=D\;f_{s}$ samples long *l*th segment, where $\omega_{\kappa },\;\kappa =0,\;1,\;\cdots ,\;Q-1$ are the frequency samples with Q=4L. Specifically, the frequency sample $\omega_{\kappa }$ that corresponds to the maximum periodogram value is extracted as a first ENF estimate. Afterward, a quadratic interpolation is employed to obtain a refined ENF estimate.

ESPRIT is also employed to estimate the ENF signal. Let $\hat{R}$ be the sample covariance matrix

(5) $$\hat{R}=\frac{1}{L}\sum_{t=m}^{L}\tilde{x}\left(t\right){\tilde{x}}^{⊤}\left(t\right)$$

where ${}^{⊤}$ stands for transposition and

(6) $$\tilde{x}\left(t\right)≜{\left[x\left(t\right),\;x\left(t-1\right),\;\ldots ,\;x\left(t-m+1\right)\right]}^{⊤}.$$

Let $\hat{S}$ be the subspace spanned by the *W* principal eigenvectors of $\hat{R}$. Let ${\hat{S}}_{1}=\left[I_{m-1}|0\right]\;\hat{S}$ and ${\hat{S}}_{2}=\left[0|I_{m-1}\right]\;\hat{S}$, where $I_{m-1}$ denotes the (m−1)×(m−1) identity matrix. ESPRIT estimates the angular frequencies ${\left\{\omega_{\kappa }\right\}}_{\kappa =1}^{W}$ as $-\arg({\hat{v}}_{\kappa })$, where ${\left\{{\hat{v}}_{\kappa }\right\}}_{\kappa =1}^{W}$ are the eigenvalues of the estimated matrix $\hat{\phi }$ [48]:

(7) $$\hat{\phi }={\left({\hat{S}}_{1}^{⊤}{\hat{S}}_{1}\right)}^{-1}\;{\hat{S}}_{1}^{⊤}{\hat{S}}_{2}$$

The frequency $-\frac{1}{2\pi }\arg\left({\hat{v}}_{\kappa }\right)\;f_{s}$ (in Hz), which is closest to the aliased base frequency is the ENF estimate. Here, m=10 and W=3.

The proposed approach combines the generation of the mean intensity time-series x(t) with either the ESPRIT or the STFT method. An outlook of the proposed approach is depicted in Algorithm 1.
**Algorithm 1:** Proposed SLIC-based approach for ENF estimation in video recordings.**Inputs:** Number of video frames Λ, number of superpixels *N*, threshold τ, cut-off frequencies, segment duration *L*, number of overlapping segments *V*, ESPRIT parameters *m* and *W*, and reference ground truth.
**Output:** Estimated ENF vector f.
1Perform SLIC in the first frame of the video recording to generate *N* regions of similar characteristics and luminance, i.e., superpixels.2Compute mean intensity values $\zeta_{n}\left(1\right)$ of each generated region.3The mean intensity values of regions exceeding threshold τ in the computation of $x_{1}$.4Locate the generated regions in the Λ−1 remaining frames and repeat steps 2-3 to compute x(t), t=2,3,⋯,Λ.5Having computed the 1-D time-series x(t), x(t) is bandpass filtered using the cut-off frequencies described in Section 2.1.6The filtered signal is split into *V* overlapping segments. Each segment is obtained by multiplying the filtered signal with an *L*-size rectangular window. Any segment is shifted from its immediate predecessor segment by *S* s.7In each segment, the prevalent frequency derived by the ESPRIT method is employed as the ENF estimate. In the case of STFT, the frequency that corresponds to the maximum periodogram value is extracted as the ENF estimate.8Compute the MCC between the estimated ENF and the reference ground truth.


### Evaluation Metric

Having estimated ENF, a matching procedure is applied in order to objectively assess estimation accuracy. Having calculated the reference ENF captured by power mains, the MCC [49] is used to compare the estimated ENF from video recordings against the reference one. Let $f={\left[f_{1},f_{2},\cdots ,f_{K}\right]}^{⊤}$ be the estimated ENF signal at each second. Let also $g={\left[g_{1},g_{2},\cdots ,g_{\tilde{K}}\right]}^{⊤}$ for $\tilde{K}>K$ be the reference ground truth ENF, which is known, and $\tilde{g}\left(p\right)={\left[g_{p},g_{p+1},\cdots ,g_{p+K-1}\right]}^{⊤}$ be a segment of g starting at *p*. The following index is determined:

(8) $$p_{opt}=\underset{p}{\mathrm{argmax}}c\left(p\right)$$

where $p=1,2,\cdots ,\tilde{K}-K+1$ and c(p) is the sample correlation coefficient between f and $\tilde{g}\left(p\right)$ defined as:

(9) $$c\left(p\right)=\frac{f^{⊤}\tilde{g}\left(p\right)}{{∥f∥}_{2}{∥\tilde{g}\left(p\right)∥}_{2}}.$$

In Section 4.9, Fisher’s transformation was employed to assess whether the pairwise differences between the MCC delivered by the proposed approach and that of state-of-the-art one are statistically significant at a significance level of 5%.

## 4. Results

The estimation of the ENF signal is significantly affected by the nature of video recordings. In static videos, ENF presence is not affected and, thus, estimation accuracy is much higher than that in non-static videos. There, continuous motion hinders ENF estimation accuracy. Many approaches aim at overcoming this difficulty. For this reason, the state-of-the-art approach for ENF estimation in video [3], which employs intensity averaging with the Multiple Signal Classification (MUSIC) method, examines whether the video to be analyzed is a static or a non-static one. For brevity, from now on, the state-of-the-art [3] approach for both static and non-static videos will be referred to as MUSIC. The proposed approach employs either ESPRIT or STFT after SLIC. The novelty of the proposed approach lies in the fact that CCD sensors capture a time snapshot using a global shutter mechanism, which makes the distinction between static and non-static video obsolete. Thus, the proposed approach is applied regardless of whether the video recording is a static or a non-static one. It is tested on six video recordings of escalating difficulty from the publicly available dataset [50]. These recordings are either static and non-static ones. A reference ground truth signal is also available. The results are compared to those obtained by MUSIC [3]. The video recordings of the dataset employed in the paper are publicly available (https://zenodo.org/record/3549379#.YUIK7bgzaUl, accessed in 8 September 2021).

### 4.1. Dataset

Six different video recordings were recorded in Vigo, Spain, at a nominal ENF 50 Hz. Two different cameras were employed, namely, a GOPRO Hero 4 Black and an NK AC3061-4KN without an anti-flicker filter [50]. The video recordings are named as ${\mathrm{mov}}_{i},i=1,\;2,\;3,\;4,\;5,\;6$ and their types are listed in Table 2.

**Figure 1:**
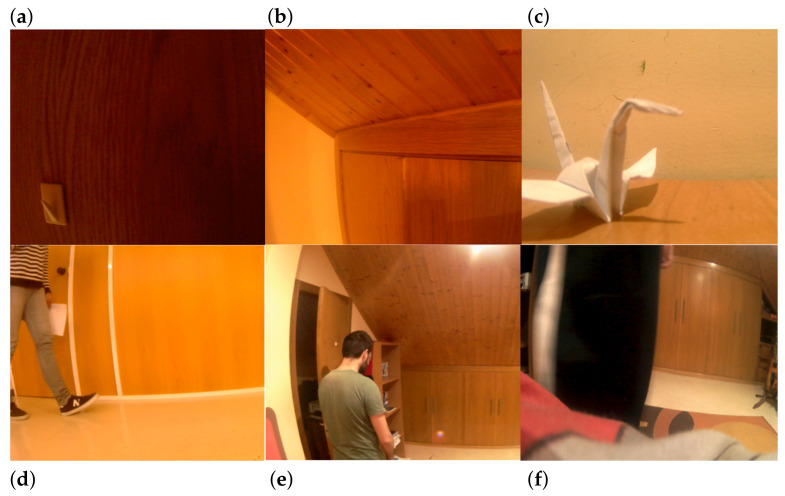
Sample frames of the six video recordings employed. (**a**) On the top left, there is a snapshot of a static video, recording a dark wall, while (**b**) on the top middle there is a snapshot of a static video, which captures the interior of a room. (**c**) On the top right, a table is depicted on which an object is placed. (**d**) On the bottom left, a person is constantly moving in an office. (**e**) On the bottom middle, there is a room with different textures and a person is moving covering a large part of the camera field many times. (**f**) On the bottom right, a person is moving in front of the camera lens inside a room.

Recording ${\mathrm{mov}}_{1}$ is closer to what is known as “white wall” video in the literature. Going a step further, it depicts a flat colored wall of low brightness. This kind of recording can be exploited to evaluate whether ENF variations can be embedded and, subsequently, estimated in such a static and seemingly noise-free environment. ${\mathrm{mov}}_{2}$ is also a static video, which contains regions with different textures, brightness, and shadows. This video is more challenging than ${\mathrm{mov}}_{1}$. ${\mathrm{mov}}_{3}$ can be categorized as a non-static video. It starts showing a white wall and a wooden table. Then, an object is placed on the table and a human hand rapidly shakes white papers at regular intervals on the right region of the recording. ${\mathrm{mov}}_{4}$ is a non-static video, where human movement appears. It is a complex recording and consists of several textures. It takes place within an office, where a human is constantly moving. Both the background wall and the floor are captured. ${\mathrm{mov}}_{5}$ constitutes one of the most challenging recordings, which resembles a real-life scene captured by a security camera. It is recorded within the complex environment of a room. The scene contains several objects with different colors and textures. The most significant challenge of ${\mathrm{mov}}_{5}$ is that the movement affects the majority of the frames and more than 50% of the pixels of each frame. ${\mathrm{mov}}_{6}$ represents another challenging video recording, which contains a constant movement of a person inside a room. The movement takes place close to the camera, affecting most pixels in each frame. In all cases, the camera is fixed. Sample frames of the video recordings are depicted in Figure 1. The estimated ENF signal is compared against a reference ground truth obtained from power mains.

### 4.2. Experimental Evaluation

The approach detailed in Section 2.1 was applied to the six video recordings and the estimated ENF was compared against the MUSIC [3] for static and non-static videos. Particularly, for static videos, the state-of-the-art approach [3] suggests averaging intensity values in each frame, while for non-static videos, intensity values are averaged within relatively static regions of each frame. In all comparisons, a rectangular temporal window was employed. The predefined threshold τ was set at MV/3, where MV is the median of *N* average intensity values within the generated regions in each frame. All approaches were implemented in MATLAB 2016a. A 64-bit operating system with an Intel(R) Core(TM) i7−5930K CPU at 3.5 GHz was used in the experiments conducted.

### 4.3. ENF Estimation in Static Video ${\mathrm{mov}}_{1}$

**Figure 2:**
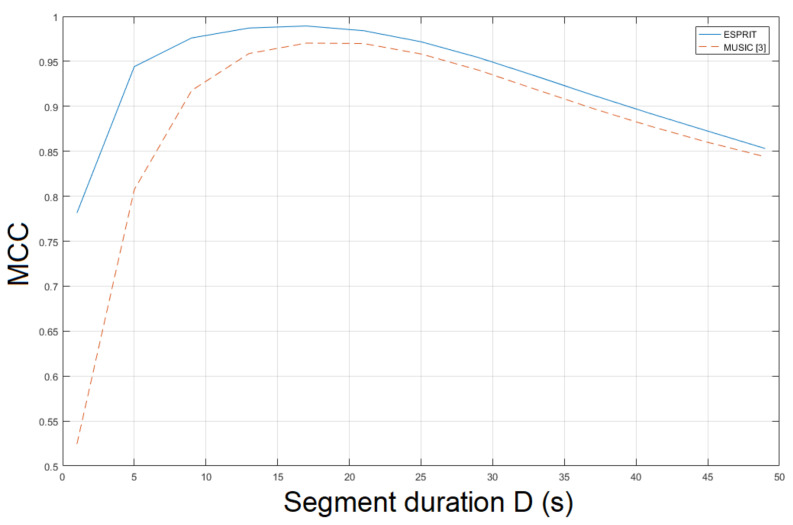
Maximum correlation coefficient of the proposed approach employing SLIC+ESPRIT for various segment durations against the MUSIC [3] (mov1).

**Figure 3:**
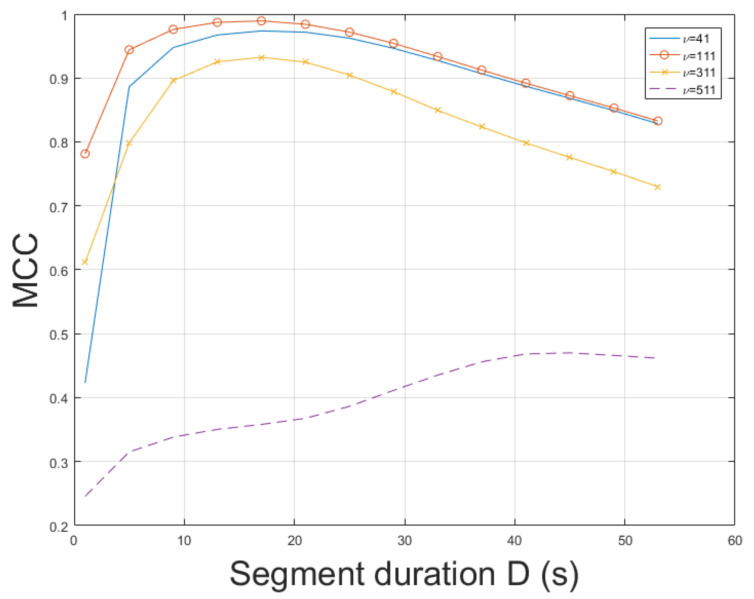
Maximum correlation coefficient of the proposed approach employing SLIC+ESPRIT for various filter orders and segment durations (mov1).

The ESPRIT method was tested for ENF estimation in ${\mathrm{mov}}_{1}$. The static nature of ${\mathrm{mov}}_{1}$ enables an accurate ENF estimation. The proposed approach, which employs the SLIC-based segmentation and intensity averaging resulted in an MCC of 0.9926, outperforming the MUSIC [3] where the MCC was measured to be 0.9658. When STFT was employed, the MCC was found to be 0.8662. Different segment durations in ENF estimation affect the results obtained. The MCC was computed for various segment durations *D*, as depicted in Figure 2. When a segment duration of 1 s was employed, the proposed approach using the ESPRIT worked satisfactorily, yielding an MCC of about 0.79, while the MCC was measured to be about 0.5, when the MUSIC [3] was used. The performance of ENF estimation depends also on the filter order ν of the bandpass filter. The MCC is plotted versus various filter orders in Figure 3. The top performance of the proposed approach, employing the ESPRIT, is achieved when ν=111. Despite ${\mathrm{mov}}_{1}$ is a trivial recording, the proposed approach offers significant improvements in ENF estimation accuracy against the method in [3]. The computational time of the proposed approach employing SLIC+ESPRIT was about 506.8 s, while the MUSIC [3] required about 492.5 s.

### 4.4. ENF Estimation in Static Video ${\mathrm{mov}}_{2}$

**Figure 4:**
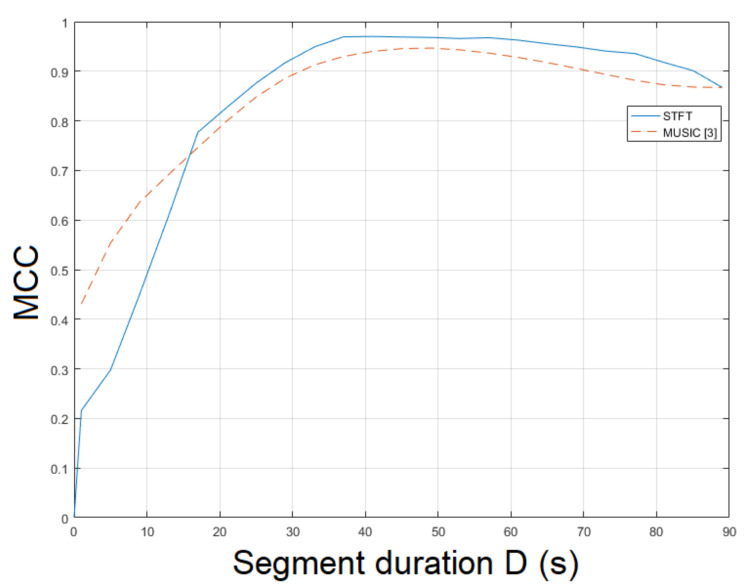
Maximum correlation coefficient of the proposed approach employing SLIC+STFT for various segment durations against the MUSIC [3] (mov2).

**Figure 5:**
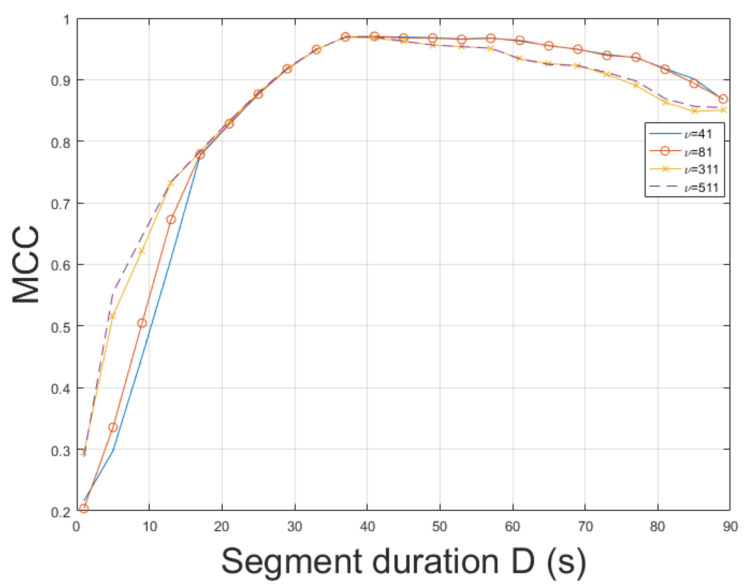
Maximum correlation coefficient of the proposed approach employing SLIC+STFT for various bandpass filter orders and segment durations (mov2).

The static recording ${\mathrm{mov}}_{2}$ is more challenging than ${\mathrm{mov}}_{1}$ due to different textures and various levels of luminance. The STFT was employed for ENF estimation yielding an MCC of 0.9704. The MUSIC [3] resulted in an MCC of 0.9466. The ESPRIT method achieved an MCC of 0.9526. In this case, there is a strong correlation between the proposed approach and the method in [3] w.r.t. segment duration. Smaller segment durations resulted in lower MCCs in both approaches. For longer segment durations, both approaches yielded a higher MCC, as shown in Figure 4. Similar behavior was noticed when different filter orders were employed. When the bandpass filter order ν=81 was used, the top performance was observed. The MCC of the proposed approach employing SLIC+STFT for various values of bandpass filter order and segment duration is plotted in Figure 5. The proposed approach employing SLIC+STFT required about 627.2 s. The computational time of the MUSIC [3] one was approximately 704.7 s.

### 4.5. ENF Estimation in Non-Static Video ${\mathrm{mov}}_{3}$

**Figure 6:**
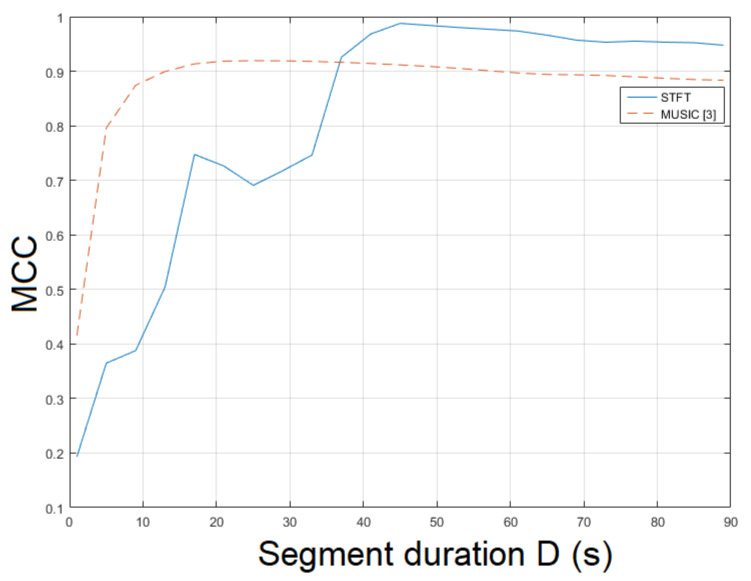
Maximum correlation coefficient of the proposed approach employing SLIC+STFT for various segment durations against the MUSIC [3] (mov3).

**Figure 7:**
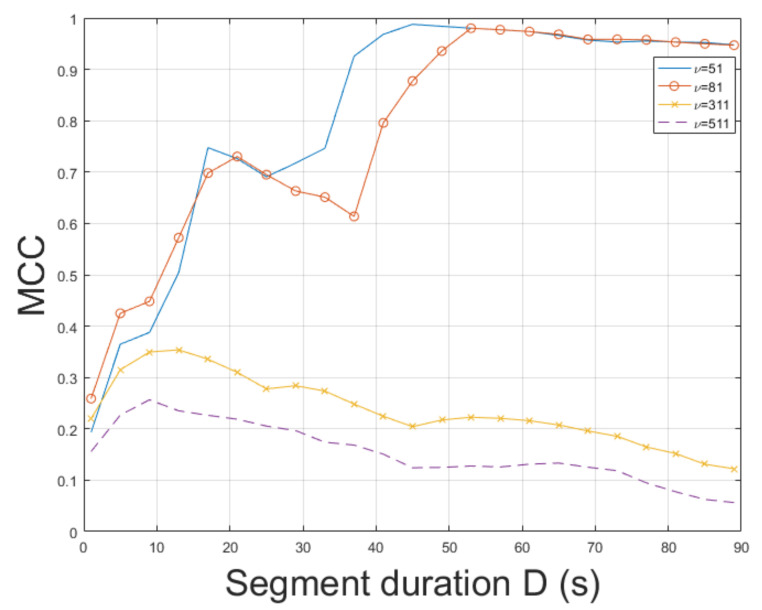
Maximum correlation coefficient of the proposed approach employing SLIC+STFT for various bandpass filter orders and segment durations (mov3).

The STFT method was employed for ENF estimation. ${\mathrm{mov}}_{3}$ is a challenging video depicting movements and different textures. Thus, ENF estimation is a non-trivial task. The STFT achieved an MCC of 0.9877, outperforming the method in [3], which reached an MCC of 0.9191. The ESPRIT method resulted in an MCC of 0.7271. As can be seen in Figure 6, the longer the segment duration, the more accurate the ENF estimation. The top result w.r.t. the MCC was measured for bandpass filter order ν=51. In ${\mathrm{mov}}_{3}$, improper values of filter order can lead to a significant reduction in MCC. Increasing the segment duration usually results in a more accurate ENF estimation w.r.t. the MCC. In this experiment, it has been noticed that when a large value of bandpass filter order is employed, increasing segment duration deteriorates estimation accuracy. The impact of filter order in MCC is demonstrated in Figure 7. The computational time of the proposed approach employing SLIC+STFT was about 468.5 s, while the MUSIC [3] required 531.4 s.

### 4.6. ENF Estimation in Non-Static Video ${\mathrm{mov}}_{4}$

**Figure 8:**
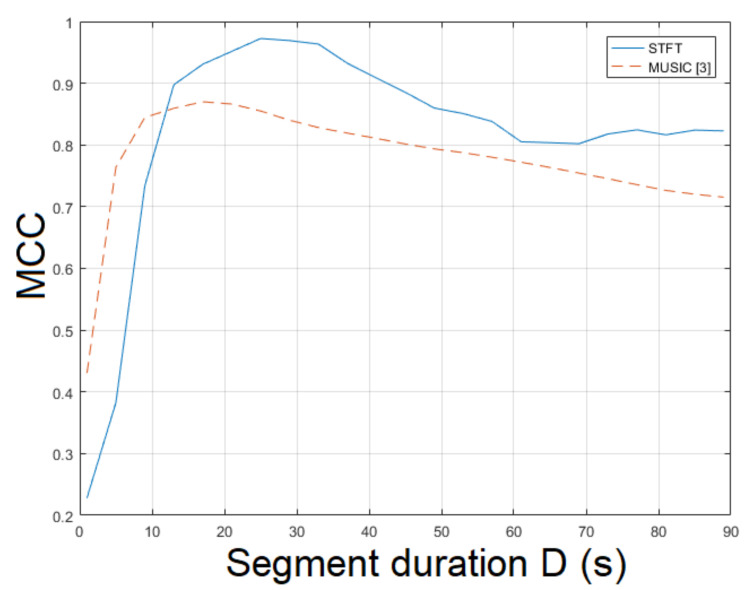
Maximum correlation coefficient of the proposed approach employing SLIC+STFT for various segment durations against the MUSIC [3] (mov4).

**Figure 9:**
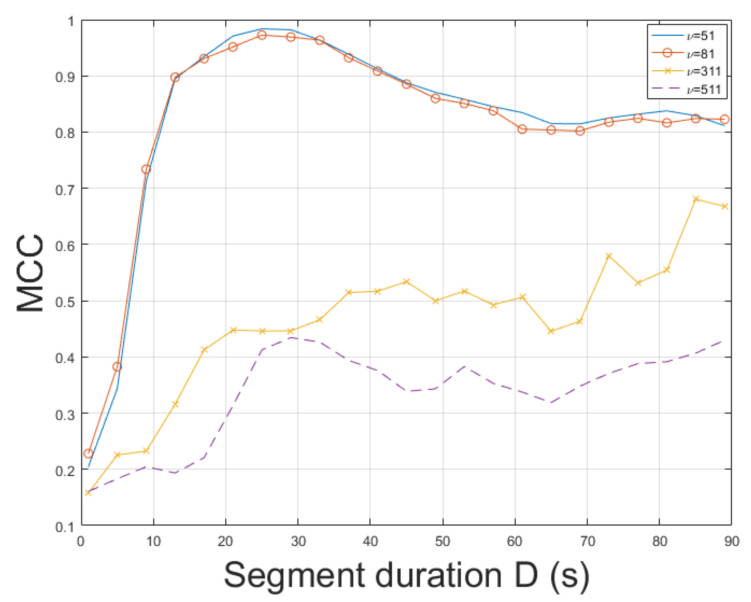
Maximum correlation coefficient of the proposed approach employing SLIC+STFT for various bandpass filter orders and segment durations (mov4).

The non-static video ${\mathrm{mov}}_{4}$ captures a much more complex scene, where the human presence and movement is closer to real-life applications than the previous videos. Here, the STFT was employed for ENF estimation. The STFT yielded an MCC of 0.9837, which outperformed the MUSIC, which attained 0.8700[3]. When the ESPRIT method was used, an MCC of 0.7605 was measured. The top performance was achieved for ν=51. The MCC of the proposed approach employing SLIC+STFT for various segment durations is shown in Figure 8. MCC values of different segment durations and various bandpass filter orders are plotted in Figure 9. The computational time required by the proposed method employing SLIC+STFT was about 423.3 s, while the execution of the MUSIC [3] required 487.2 s to conclude.

### 4.7. ENF Estimation in Non-Static Video ${\mathrm{mov}}_{5}$

**Figure 10:**
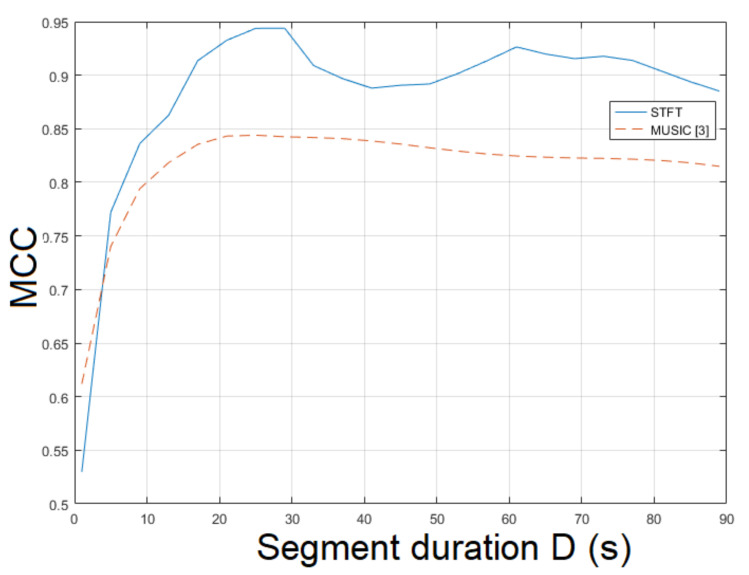
Maximum correlation coefficient of the proposed approach employing SLIC+STFT for various segment durations against the MUSIC [3] (mov5).

**Figure 11:**
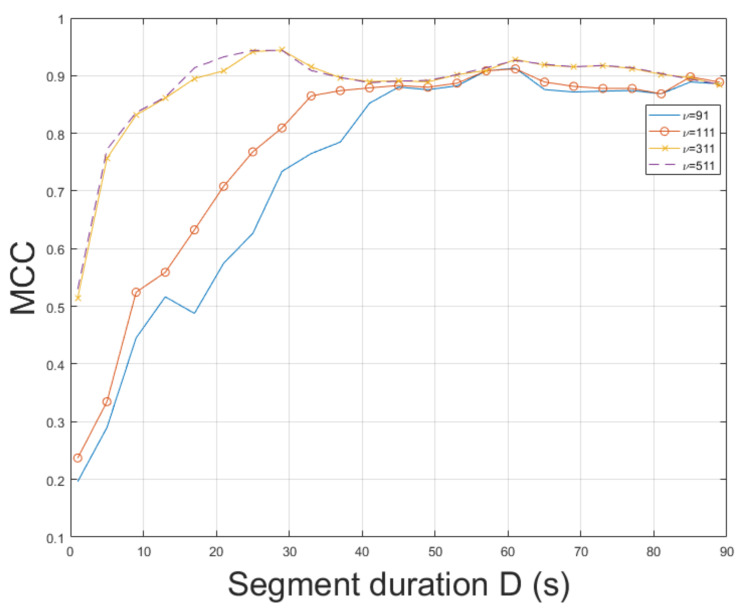
Maximum correlation coefficient of the proposed approach employing SLIC+STFT for various bandpass filter orders and segment durations (mov5).

Video ${\mathrm{mov}}_{5}$ is one of the most challenging recordings. It resembles a scene captured by a security camera. Here, the STFT was employed for ENF estimation. The STFT achieved an MCC of 0.9432, outperforming the MUSIC [3] whose MCC was measured to be 0.8441 [3]. When the ESPRIT was employed, the MCC reached 0.8959. The MCC of STFT is plotted for various segment durations against the MUSIC [3] in Figure 10. When different values of bandpass filter order were employed, a longer segment duration was found to yield an increase in MCC, as can be seen in Figure 11. On the contrary, for a segment duration longer than or equal to 40, a plateau is noticed. The top MCC was achieved for a bandpass filter order of ν=511. The execution of the proposed approach employing SLIC+STFT required 523.4 s to conclude, while the computational time of the MUSIC [3] was about 602.6 s.

### 4.8. ENF Estimation in Non-Static Video ${\mathrm{mov}}_{6}$

**Figure 12:**
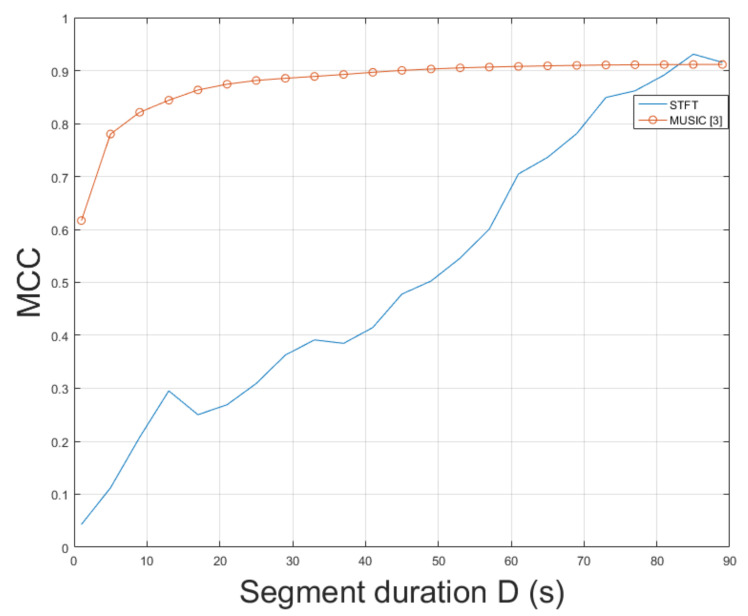
Maximum correlation coefficient of the proposed approach employing SLIC+STFT for various segment durations against the MUSIC [3] (mov6).

**Figure 13:**
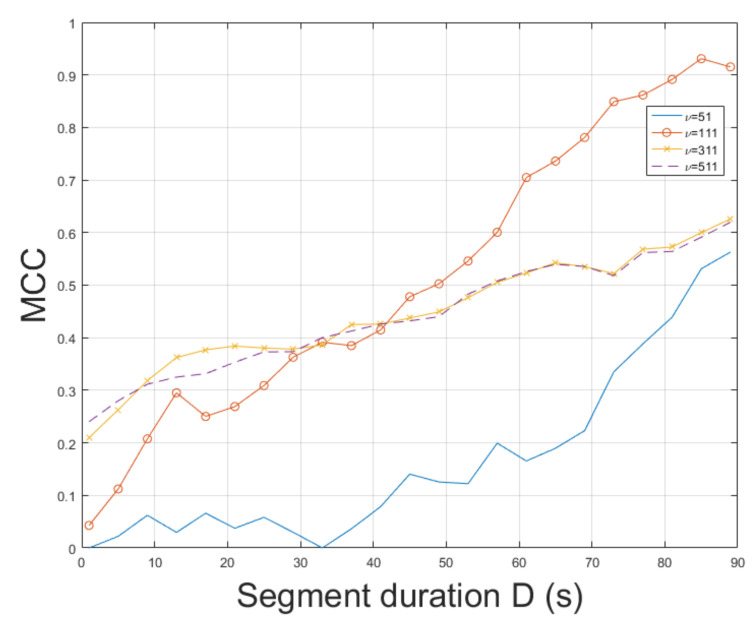
Maximum correlation coefficient of the proposed approach employing SLIC+STFT for various bandpass filter orders and segment durations (mov6).

Similarly to video ${\mathrm{mov}}_{5}$, ${\mathrm{mov}}_{6}$ constitutes a challenging real-world indoor recording. This recording resembles a scene captured by a hidden camera under special conditions, which could hinder ENF estimation accuracy. Nevertheless, the proposed approach employing STFT resulted in an MCC of 0.9309, outperforming the MUSIC [3] whose MCC was measured to be 0.9115. The MCC of SLIC+STFT is plotted for various segment durations against the MUSIC [3] in Figure 12. The proposed approach performs better than the MUSIC [3] for a segment duration of about 85 s. For shorter segment durations, the MUSIC [3] demonstrates a stable performance, outperforming the proposed SLIC+STFT. For different values of bandpass filter order, it is worth mentioning that by increasing segment duration, an increase in MCC is observed for all cases, as can be seen in Figure 13. The top MCC was achieved for a bandpass filter order of ν=111. The execution of the proposed approach was 572.5 s. The execution of the MUSIC [3] method required 639.5 s to conclude.

### 4.9. Assessment of MCC Differences

In order to assess whether the improvements in MCC of the proposed approach, employing SLIC and either STFT or ESPRIT against the MUSIC [3] is statistically significant, and hypothesis testing was applied to all six recordings. The null hypothesis, $H_{0}$: $c_{1}=c_{2}$, indicates that MCCs are equal and the alternative one, $H_{1}$: $c_{1}\neq c_{2}$ indicates the opposite.

For each video recording, the MCCs of the proposed approach and the MUSIC [3] undergo Fisher’s *z* transformation [51]:

(10) $$z=0.5\ln\frac{1+c}{1-c}.$$

The test statistic is given by:

(11) $$q_{F}=\sqrt{K-3}\;\left(z_{1}-z_{2}\right)$$

where *K* denotes the number of ENF samples. The test statistic $q_{F}$ is distributed as Gaussian with zero mean value and unit variance, for large *K*.

It is checked whether the test statistic $q_{F}$ falls within the region of acceptance for a significance level of 5%. If it does so, the null hypothesis $H_{0}$ is accepted and, thus, the differences between the MCC’s are not statistically significant. On the other hand, if $q_{F}$ falls outside the region of acceptance (i.e., $|q_{F}|>1.965$), the alternative hypothesis $H_{1}$ is accepted, indicating that MCC differences are statistically significant. Statistical tests constitute an important contribution of the paper, offering a mechanism for making quantitative decisions, which can lead to accurate ENF estimation in practical forensic applications. The top MCC value of the proposed approach employing SLIC and either STFT or ESPRIT and that of the MUSIC [3] for each recording and the filter order employed is summarized in Table 3.

In all cases in Table 3, $q_{F}$ was calculated and found to be outside the region of acceptance for significance level of 5%. Consequently, there is sufficient evidence to warrant the rejection of the null hypothesis. Therefore, the differences between the MCCs are statistically significant and the proposed approach yields statistically significant improvements in ENF estimation accuracy against the MUSIC [3].

## 5. Conclusions, Limitations, and Future Research

ENF estimation in static and non-static videos is a non-trivial task especially for complex environments comprising different objects, textures, and moving people. A novel automated approach has been proposed for ENF estimation in static and non-static videos recorded with CCD sensors. It is based on the SLIC algorithm for the generation of regions that share similar characteristics, especially luminance, where ENF variations can be precisely revealed. It has been demonstrated that the proposed approach, which applies either STFT or ESPRIT to a time-series created after SLIC, performs better than the MUSIC [3] in ENF estimation with respect to the maximum correlation coefficient. Moreover, the impact of two factors, namely, the segment duration and the bandpass filter order in ENF estimation accuracy, has been studied. Statistical tests have been conducted, attesting that the improvements in maximum correlation coefficient achieved by the proposed approach are statistically significant against the state-of-the-art approach, which employs the MUSIC method.

In this work, we have explored multiple videos recorded by a fixed camera. A scenario with a moving camera would possibly raise additional difficulties in finding areas of similar characteristics, which are employed in the proposed approach. Consequently, difficulties in accurately estimating the ENF estimate would be anticipated. In addition, although the recordings were of escalating difficulty, there was no more than one person present in the scene. It is difficult to predict whether the proposed approach would perform equally well in an unconstrained environment with a moving camera and scenes with many moving persons.

Future work will aim to extend this work by considering recordings that are captured by the rolling shutter mechanism of CMOS cameras. We are also interested in ENF estimation, when non-static cameras are employed. The latter scenario is very common in real-life applications due to the widespread use of mobile phones. Another challenging research direction is ENF estimation when multiple persons are recorded in the video.

## Figures and Tables

**Table 1:** Aliased frequencies of ENF with respect to (w.r.t.) camera frame rate and fundamental ENF at power mains frequency [3].

Power Mains (Hz)	Camera Frame Rate $f_{s}$ (fps)	Aliased Base Frequency (Hz)
50	29.97	10.09
50	30	10
60	29.97	0.12
60	30	0

**Table 2:** Types of six video recordings employed for ENF estimation.

Video Name	Video Type
${\mathrm{mov}}_{1}$	static
${\mathrm{mov}}_{2}$	static
${\mathrm{mov}}_{3}$	non-static
${\mathrm{mov}}_{4}$	non-static
${\mathrm{mov}}_{5}$	non-static
${\mathrm{mov}}_{6}$	non-static

**Table 3:** Maximum correlation coefficient of the proposed approach employing either STFT or ESPRIT and the MUSIC [3] for all recordings. The filter order employed is also quoted.

mov	MCC (here)	MCC [3]	Bandpass Filter Order ν	ENF Samples *K*
${\mathrm{Mov}}_{1}$	0.9926	0.9658	111	702
${\mathrm{Mov}}_{2}$	0.9704	0.9466	81	639
${\mathrm{Mov}}_{3}$	0.9877	0.9191	51	647
${\mathrm{Mov}}_{4}$	0.9837	0.8700	51	623
${\mathrm{Mov}}_{5}$	0.9432	0.8441	511	729
${\mathrm{Mov}}_{6}$	0.9309	0.9115	111	741

## Data Availability

The data presented in this study are openly available in Zenodo at https://doi.org/10.5281/zenodo.3549379, accessed on 8 September 2021.

## Abbreviations

f	ENF signal
Hz	Hertz
g	reference ENF signal
ν	filter order
$f_{s}$	sampling frequency of camera (in frames per second (fps))
$f_{E}$	aliased frequency
$f_{l}$	frequency of light source illumination
I	identity matrix
γ	integer number
x(t)	mean intensity signal
${\hat{\phi }}_{l}$	periodogram
*D*	segment duration (in s)
*L*	segment duration (in samples)
*G*	hop size (in samples)
m	order of the covariance matrix
τ	predefined threshold
*S*	segment shift (in s)
MV	median intensity value
*N*	number of superpixels
*K*	number of estimated ENF values
Λ	number of video frames
$\zeta_{n}$	mean intensity value
*V*	overlapping segments
$q_{F}$	test statistic
W	principal eigenvectors
CCD	charge-coupled device
CMOS	complementary metal oxide semiconductor
ENF	electric network frequency
ESPRIT	estimation by rotational invariant techniques
MCC	maximum correlation coefficient
MUSIC	multiple signal classification
STFT	short-time Fourier transform
SLIC	simple linear iterative clustering

## References

[1] Castillo Camacho I., Wang K. (2021). A Comprehensive Review of Deep-Learning-Based Methods for Image Forensics. J. Imaging.

[2] Grigoras C. (2005). Digital audio recording analysis: The electric network frequency (ENF) criterion. Int. J. Speech Lang. Law.

[3] Garg R., Varna A.L., Hajj-Ahmad A., Wu M. (2013). “Seeing” ENF: Power-Signature-Based Timestamp for Digital Multimedia via Optical Sensing and Signal Processing. IEEE Trans. Inf. Forensics Secur..

[4] Hua G., Liao H., Wang Q., Zhang H., Ye D. (2020). Detection of Electric Network Frequency in Audio Recordings—From Theory to Practical Detectors. IEEE Trans. Inf. Forensics Secur..

[5] Ojowu O., Karlsson J., Li J., Liu Y. (2012). ENF Extraction from Digital Recordings Using Adaptive Techniques and Frequency Tracking. IEEE Trans. Inf. Forensics Secur..

[6] Bykhovsky D., Cohen A. (2013). Electrical Network Frequency (ENF) Maximum-Likelihood Estimation via a Multitone Harmonic Model. IEEE Trans. Inf. Forensics Secur..

[7] Hajj-Ahmad A., Garg R., Wu M. (2013). Spectrum Combining for ENF Signal Estimation. IEEE Signal Process. Lett..

[8] Lin X., Kang X. (2018). Robust Electric Network Frequency Estimation with Rank Reduction and Linear Prediction. ACM Trans. Multimed. Com. Commun. Appl..

[9] Karantaidis G., Kotropoulos C. (2021). Blackman–Tukey spectral estimation and electric network frequency matching from power mains and speech recordings. IET Signal Process..

[10] Fu L., Markham P.N., Conners R.W., Liu Y. (2013). An Improved Discrete Fourier Transform-Based Algorithm for Electric Network Frequency Extraction. IEEE Trans. Inf. Forensics Secur..

[11] Karantaidis G., Kotropoulos C. Assessing spectral estimation methods for Electric Network Frequency extraction. Proceedings of the 22nd Pan-Hellenic Conference on Informatics.

[12] Karantaidis G., Kotropoulos C. Efficient Capon-Based Approach Exploiting Temporal Windowing for Electric Network Frequency Estimation. Proceedings of the 2019 IEEE 29th International Workshop on Machine Learning for Signal Processing (MLSP).

[13] Dosiek L. (2015). Extracting Electrical Network Frequency From Digital Recordings Using Frequency Demodulation. IEEE Signal Process. Lett..

[14] Hajj-Ahmad A., Garg R., Wu M. Instantaneous frequency estimation and localization for ENF signals. Proceedings of the 2012 Asia Pacific Signal and Information Processing Association Annual Summit and Conference.

[15] Cooper A.J. (2008). The electric network frequency (ENF) as an aid to authenticating forensic digital audio recordings—An automated approach. Audio Engineering Society Conference: 33rd International Conference: Audio Forensics-Theory and Practice.

[16] Hua G., Liao H., Zhang H., Ye D., Ma J. (2021). Robust ENF Estimation Based on Harmonic Enhancement and Maximum Weight Clique. IEEE Trans. Inf. Forensics Secur..

[17] Zhu Q., Chen M., Wong C., Wu M. (2020). Adaptive multi-trace carving for robust frequency tracking in forensic applications. IEEE Trans. Inf. Forensics Secur..

[18] Liao H., Hua G., Zhang H. (2021). ENF Detection in Audio Recording via Multi-Harmonic Combining. IEEE Signal Process. Lett..

[19] Esquef P.A.A., Apolinario J.A., Biscainho L.W.P. (2014). Edit Detection in Speech Recordings via Instantaneous Electric Network Frequency Variations. IEEE Trans. Inf. Forensics Secur..

[20] Rodriguez D.P.N., Apolinario J.A., Biscainho L.W.P. (2010). Audio Authenticity: Detecting ENF Discontinuity With High Precision Phase Analysis. IEEE Trans. Inf. Forensics Secur..

[21] Hua G., Zhang Y., Goh J., Thing V.L.L. (2016). Audio Authentication by Exploring the Absolute-Error-Map of ENF Signals. IEEE Trans. Inf. Forensics Secur..

[22] Reis P.M.J.I., Costa J.P.C.L., Miranda R.K., Galdo G.D. (2017). ESPRIT-Hilbert-Based Audio Tampering Detection with SVM Classifier for Forensic Analysis via Electrical Network Frequency. IEEE Trans. Inf. Forensics Secur..

[23] Garg R., Hajj-Ahmad A., Wu M. Geo-location estimation from electrical network frequency signals. Proceedings of the 2013 IEEE International Conference on Acoustics, Speech and Signal Processing.

[24] Hajj-Ahmad A., Garg R., Wu M. (2015). ENF-Based Region-of-Recording Identification for Media Signals. IEEE Trans. Inf. Forensics Secur..

[25] Lin X., Liu J., Kang X. (2016). Audio Recapture Detection With Convolutional Neural Networks. IEEE Trans. Multimed..

[26] Hua G., Zhang H. (2020). ENF Signal Enhancement in Audio Recordings. IEEE Trans. Inf. Forensics Secur..

[27] Elmesalawy M.M., Eissa M.M. (2014). New Forensic ENF Reference Database for Media Recording Authentication Based on Harmony Search Technique Using GIS and Wide Area Frequency Measurements. IEEE Trans. Inf. Forensics Secur..

[28] Bykhovsky D. (2020). Recording device identification by ENF harmonics power analysis. Forensic Sci. Int..

[29] Hajj-Ahmad A., Wong C., Gambino S., Zhu Q., Yu M., Wu M. (2019). Factors Affecting ENF Capture in Audio. IEEE Trans. Inf. Forensics Secur..

[30] Su H., Hajj-Ahmad A., Garg R., Wu M. Exploiting rolling shutter for ENF signal extraction from video. Proceedings of the 2014 IEEE International Conference on Image Processing (ICIP).

[31] Vatansever S., Dirik A.E., Memon N. (2019). Analysis of Rolling Shutter Effect on ENF-Based Video Forensics. IEEE Trans. Inf. Forensics Secur..

[32] Vatansever S., Dirik A.E., Memon N. (2017). Detecting the Presence of ENF Signal in Digital Videos: A Superpixel-Based Approach. IEEE Signal Process. Lett..

[33] Fernández-Menduiña S., Pérez-González F. (2020). Temporal Localization of Non-Static Digital Videos Using the Electrical Network Frequency. IEEE Signal Process. Lett..

[34] Hajj-Ahmad A., Berkovich A., Wu M. (2016). Exploiting power signatures for camera forensics. IEEE Signal Process. Lett..

[35] Su H., Hajj-Ahmad A., Wong C., Garg R., Wu M. ENF signal induced by power grid: A new modality for video synchronization. Proceedings of the 2nd ACM International Workshop on Immersive Media Experiences.

[36] Su H., Hajj-Ahmad A., Wu M., Oard D. Exploring the use of ENF for multimedia synchronization. Proceedings of the 2014 IEEE International Conference on Acoustics, Speech and Signal Processing (ICASSP).

[37] Wang Y., Hu Y., Liew A., Li C.T. (2020). ENF Based Video Forgery Detection Algorithm. Int. J. Digit. Crime Forensics.

[38] Nagothu D., Chen Y., Blasch E., Aved A., Zhu S. (2019). Detecting malicious false frame injection attacks on surveillance systems at the edge using electrical network frequency signals. Sensors.

[39] Wong C.W., Hajj-Ahmad A., Wu M. Invisible Geo-Location Signature in A Single Image. Proceedings of the 2018 IEEE International Conference on Acoustics, Speech and Signal Processing (ICASSP).

[40] Ferrara P., Sanchez I., Draper-Gil G., Junklewitz H., Beslay L. A MUSIC Spectrum Combining Approach for ENF-based Video Timestamping. Proceedings of the 2021 IEEE International Workshop on Biometrics and Forensics (IWBF).

[41] Achanta R., Shaji A., Smith K., Lucchi A., Fua P., Süsstrunk S. (2012). SLIC superpixels compared to state-of-the-art superpixel methods. IEEE Trans. Pattern Anal. Mach. Intell..

[42] Grigoras C. (2007). Applications of ENF criterion in forensic audio, video, computer and telecommunication analysis. Forensic Sci. Int..

[43] Nicolalde-Rodríguez D.P., Apolinário J.A., Biscainho L.W.P. Audio authenticity based on the discontinuity of ENF higher harmonics. Proceedings of the 21st European Signal Processing Conference (EUSIPCO 2013).

[44] Hu Y., Li C.T., Lv Z., Liu B.B. (2012). Audio forgery detection based on max offsets for cross correlation between ENF and reference signal. International Workshop on Digital Watermarking.

[45] Oppenheim A.V., Schafer R.W. (2009). Discrete-Time Signal Processing.

[46] Hajj-Ahmad A., Baudry S., Chupeau B., Doërr G. Flicker forensics for pirate device identification. Proceedings of the 3rd ACM Workshop on Information Hiding and Multimedia Security.

[47] Allen J.B., Rabiner L.R. (1977). A unified approach to short-time Fourier analysis and synthesis. Proc. IEEE.

[48] Stoica P., Moses R.L. (2005). Spectral Analysis of Signals.

[49] Huijbregtse M., Geradts Z. (2009). Using the ENF criterion for determining the time of recording of short digital audio recordings. International Workshop on Computational Forensics.

[50] Fernandez-Menduina S., Pérez-González F. (2020). ENF Moving Video Database.

[51] Papoulis A. (1990). Probability and Statistics.

